# Comparison of WLAN Probe and Light Sensor-Based Estimators of Bus Occupancy Using Live Deployment Data

**DOI:** 10.3390/s22114111

**Published:** 2022-05-28

**Authors:** Tatiana Madsen, Hans-Peter Schwefel, Lars Mikkelsen, Annelore Burggraf

**Affiliations:** 1 Department of Electronic Systems, Aalborg University, 9220 Aalborg, Denmark; hps@es.aau.dk; 2 Keysight AS, Alfred Nobels Vej 21E, 9200 Aalborg, Denmark; larsmikkelsen@gmail.com; 3 Wolfsburg AG MobilitätsWirtschaft, Major-Hirst-Straße 11, 38442 Wolfsburg, Germany; annelore.burggraf@wolfsburg-ag.com

**Keywords:** WLAN probes, light sensor system, public transport, automated passenger counting, bus occupancy estimation

## Abstract

Bus company operators are interested in obtaining knowledge about the number of passengers on their buses—preferably doing so at low deployment costs and in an automated manner, while keeping accuracy high. One solution, widely used in practice, involves deploying a light sensor-based system, counting the people entering and leaving the bus. The light sensor system is simple, but errors accumulate over time, because it is not capable of error correcting. For this reason, the light sensor-based system is compared to a WLAN probe-based system, which has entirely different characteristics. Inaccuracy with the WLAN estimator comes from a need to filter out mobile devices outside the bus and to map the number of detected devices to a number of people. The comparison is performed based on data collected from a real-life deployment in a medium sized German city. The comparison shows the trade-off in selecting either of the two methods. Furthermore, a novel approach for fusion of the light sensor and WLAN estimators is proposed which has a big potential in improving accuracy of both estimators. A fusion approach is proposed that utilizes the different error characteristics for error compensation by calculating compensation terms. The knowledge of Ground Truth is not required as part of this fusion approach for calibration; results show that the approach can find the optimal parameter settings and that it makes this occupancy estimation approach scalable and automated.

## 1. Introduction

Public transport users are gaining more support from traveling apps, providing travelers with travel options, routes, time schedules, and live updates. However, a final element is missing in the information on public transport, namely information about the number of travelers, i.e., the occupancy. This information has several use cases for the public transport user and provider.

Given information about the occupancy of buses, the traveler can decide to take another route or a later bus, e.g., if traveling with a group, and for that reason, the need for more seats. The traveler could also select the bus based on the occupancy simply because it is more comfortable to travel in less loaded buses. Either way, there would be less need for the traveler to know about special events or peak periods that put more load on the public transport system, as this information would be readily available.

The public transport provider could also make use of bus occupancy information. If the information is available in a close to real-time form, e.g., only delayed a few minutes, the bus company could assign more resources (buses) to a bus line in an ad-hoc manner. A current use case for collecting statistics on the number of passengers is for the bus company to claim subsidies from the local government, which is based on the number of passengers [1,2]. Thus, when the statistics are collected using measurement techniques, it is vital to obtain information about the errors of the methods.

This work compares two methods for estimating the number of passengers in public buses; one method is based on light sensors in the doors, while the other is based on WLAN probes collected from devices carried by travelers. The specific WLAN probe-based system used in this context was previously described in [3]. The comparison was conducted by evaluating the error between the ground truth passenger count and the obtained estimates. This error was calculated just after every bus stop; as the WLAN based estimator did not have knowledge about the bus stop, an approach to align this estimator to bus stops was introduced and applied.

Additionally, the changes in the number of passengers was aligned to bus stops, which results in better accuracy and enables a fair direct comparison with the light sensor-based estimator and ground truth. The goal was to assess the performance of the WLAN based approach when directly compared to an already deployed automated occupancy counting system. This evaluation was done based on data collected from the two systems deployed on buses, in a live operation, in a medium-sized German city. Finally, a fusion approach was introduced and assessed, which allowed deriving correction parameters for both approaches without the need for knowledge of ground truth.

These two specific approaches for estimating the number of passengers were chosen for a few reasons. Firstly, the light sensor based approach is the most commonly used passive approach for such estimation, and for this reason, it can be seen as the baseline system in comparison with other methods. Furthermore, this system estimates the number of passengers based on directly measuring the physical presence of passengers. The WLAN probe-based approach was chosen because it is a non-intrusive passive sensing measurement approach. Furthermore, it is a very low-cost approach in terms of hardware and deployment. Where it differs from the light sensor approach is that the WLAN probe-based approach does not directly sense the passengers, it derives the number of passengers based on the presence of WLAN-enabled devices.

Where this work differs from the related work is that a direct comparison was performed between two low-cost device-based non-intrusive passenger counting approaches. Furthermore, an analysis was conducted based on data collected from a real world deployment on live buses. The advantage of using data collected from buses in every-day operation is that real world issues and implications are evaluated, which might not be covered in a lab setup. The obtained results were subsequently used in a novel fusion approach, which led to significant accuracy improvements.

This paper is an extension of the work presented in [4], where the initial studies on the two bus occupancy estimators are presented. In this work, an extended description of the estimators and sensor systems is given, along with an in depth estimator comparison. Additionally, we took a step beyond comparing the two estimator and suggest a novel approach for estimator fusion. Observing the nature of estimation errors in both cases, we propose a mathematical model that describes bus occupancy estimation and accounts for estimation errors, by introducing correction factors. Solving the derived equation for the minimum mean squared error (MSE), the correction factors for both estimators can be found. Evaluation results show that the proposed approach can significantly improve the estimation accuracy. The advantage of the approach is that no other information (e.g., ground truth) is required to make a calibration of the estimators for unique operating conditions.

The data used for the analysis in this work are made available for other researchers as an open data set, and can be accessed at [5]. The data are anonymized, both in terms of device IDs (MAC addresses) and locations, by conversion of GPS coordinates into Cartesian coordinates with an arbitrarily chosen origin point. The data include data collected from the light sensor system, the WLAN probe sensor system, ground truth data manually obtained on the bus trips, and information about the location of bus stops and bus stop sequences (bus routes).

The rest of the paper is structured as follows: Section 2 presents an overview of the different types of people count approaches. Section 3 presents the data sets that the analysis and comparison are based on. Section 4 briefly presents the system for collecting WLAN probes and introduces the WLAN probe-based estimator, including extrapolation from devices to the number of people. In Section 5 and Section 6, the WLAN probe-based estimator is aligned to match bus stops, and estimator parameters are selected. Section 7 describes the light sensor system and the estimator based on this, including evaluation of the error probability. In Section 8, the two estimators are compared using ground truth data. Section 9 presents the improved estimation by fusion of the information obtained from the two estimators. Finally, Section 10 presents a conclusion of the findings in comparison of the estimators.

## 2. Related Work

Several different approaches exists to obtain information about the number of people, the number of passengers, or footfall count. In this work, the focus is on estimating the number of passengers on public transport, and buses in particular. In [6], the authors distinguish between three categories for counting people; namely video-based, device-free non-image based, and device-based non-image based recognition. In the following, different technologies and approaches within these categories will be discussed in the setting of estimating the number of passengers on a bus.

The current setting, in terms of public transport, imposes some requirements to the solution, such as providing a highly accurate “people count” within a small spatial area (a bus), as well as providing an estimated number (in close to real-time) due to the dynamics of passengers entering and exiting buses.

In [7], the authors performed real-time passenger flow (in and out of the bus) estimations based on analyses of images from a single camera on a bus. In this approach, the images were analyzed to recognize outlines of passenger heads. The approach showed high accuracy from test results, but also displayed issues concerning certain combinations of hair color and background light and color. In [8], a more advanced stereoscopic camera is utilized, which offers a better depth perception than the traditional single camera setup. The additional lens allows the algorithm to utilize additional features when processing the images, which shows good results with high accuracy when test data are evaluated. Moreover, this approach “struggles” with varying light settings in the images. A common limitation for image-based approaches is that they require a line of sight to passengers in order to count them. In addition, the use of cameras can raise privacy concerns for passengers.

Device-free passenger counting approaches require that some sensing be done aboard the bus. One such approach is described in [9], where information extracted from an IC card system, i.e., a card with a chip that passengers use to pay for public transport trips. This covers information about where passengers traveled to and from, and when. The assumption in this paper is that all passengers need to both check in and check out with their card; these check-in and check-out events then allow to determine the number of passengers on the bus at any given time. Based on this the accumulated number of passengers at a given time can be extracted. The authors use the extracted information to create a forecast of passenger flows. Another similar approach is described in [10], where information about individual passenger trips is extracted from an automated fare collection system, i.e., where the passenger travels to and from, and when. Here, the information is used to predict passenger flows of public transport. Information about passenger presence obtained online from an electronic ticketing system is the most accurate form of passenger estimation in real-time. However, if this information is available only offline, prediction of future passenger flow is necessary, which makes this approach vulnerable to unprecedented spikes in traveler numbers, e.g., in connection with citywide events. However, this depends on the specific implementation and the willingness of the transport operator to make this information available in close to real-time.

Other device-free approaches rely on various sensor types located on the bus. One example of this is presented in [11], where sensors are integrated in the bus seats to detect whether passengers occupy them or not. This approach provides a high accuracy of real-time passenger estimation, but the downside is that a sensor must be installed in each seat. Another sensor-based approach involves utilizing light sensors located in bus doors, as described in [12]. The system senses people passing through doors, which, when accumulated, can yield information about the current number of passengers on the bus. The system provides a high accuracy, and for that reason it is one of the most common approaches used to count passengers. A drawback of the light sensor-based system is that doors must be strictly used for entrance or exit. If a door can be used for both, the light sensor system cannot easily identify whether passengers are entering or exiting when passing though the sensor area.

In the device-based category, several approaches exist for counting people that utilize different technologies. These can generally be divided into active and passive approaches, or into intrusive and non-intrusive approaches. Active device-based approaches means that devices actively provide specialized information to a centralized system. One example of this is described in [13], where a crowdsourcing system is developed and utilized for collecting information about passenger count. The user of the device provides feedback about number of passengers on the bus by entering the information in the system. This approach requires that a person participating in the crowdsourcing system is present on the bus to obtain information about passenger count. Furthermore, to have updated close to real-time information, the users must continuously report the passenger count to the system. Another active device-based approach is described in [6], where a mesh of devices are utilized to collect information about the presence of other devices. The information is collected, centralized, and analyzed to figure out if devices are in the vicinity of many or a few other devices. Based on this, the people density can be estimated in different locations. This approach has a similar weakness as the previous, in that the presence of specialized devices are required in order to estimate the number of people.

The last type of passenger-estimation approaches are device-based passive approaches that opportunistically collect information about devices, based on communication already generated from the devices in other contexts. One example of this approach is described in [14], where information is extracted from a cellular network, about which devices are connected to which cell towers. With this information, along with knowledge of the cell tower locations, the footfall in a cell grid is estimated. It was demonstrated that the accuracy, in terms of assigning devices to the right cell, depends on the definition of the grid structure in relation to the cell tower locations. Tweaking this leads to high accuracy, but only within individual cells of the grid, meaning that the spatial resolution is low. Furthermore, the approach is also not able to distinguish whether a device is carried by a person in a bus or walking on the street.

Another example of this approach is based on the collection of WLAN probes. This approach exploits a feature in WLAN where devices broadcast probe requests, containing the MAC address of the device, to discover access points in the vicinity. The probes are collected passively by a sensor, and based on this information, it is possible to count devices in the vicinity, and based on the received signal strength the proximity of each device can be evaluated. The feasibility of this approach is investigated in [15], focusing on the correlation between the received signal strength and distance to devices. They also propose a method for obtaining a factor to get from the number of devices to the number of people, but the sensor is placed at a bus stop. In [16], the same approach is used to count passengers on a bus, highlighting advantages of the approach, such as unaffected by bus doors being used for both entrance and exit. In [17], further experiences are presented from the WLAN probe-based approach related to system deployment, data processing algorithm, parameter selection, and general passenger estimation accuracy. Other realizations that illustrate the viability of this approach are described in [18], with the deployment of a system named Trellis in a bus system in Madison, Wisconsin. In [19], the capabilities of the WLAN based approach is demonstrated via a deployment of buses in Madrid. In [20], the approach is used in junction with Bluetooth beacons to estimate people flow through security check-in at the airport. The WLAN-based approach described in the above listed works offers non-intrusive passenger estimation in a small area, which is fitting for the current scenario. However, the approach is subject to noise or signals from devices in the vicinity that are not meant to be counted. Furthermore, the frequency of emitting probes is not fixed, which makes it difficult to say when all devices in the vicinity have been heard from. Another weakness, common for all the device-based approaches, is that the estimation is done for the devices in the area and this requires a mapping between the number of devices and the number of people, taking into account chances that a passenger carries multiple devices or does not carry any device at all.

This work differs from previous related work in that it provides an evaluation of live data from a co-deployed light sensor system and WLAN probe sensor system, which allows for an in-depth analysis and comparison of the two bus occupancy estimation approaches. The insights from the comparison lead to a novel fusion approach that significantly improves the accuracy.

## 3. Data Set Overview

Throughout this paper, a number of data sets are used, either for parameter estimation for the estimation methods or for comparison of the estimation approaches. All data sets were collected on the same bus route going from one side of a medium-sized German city, through the city center, and to the other side, i.e., each data set was collected on a single, different bus trip. The choice of the bus routes implies that the bus passes through suburban and urban environments. All data sets were collected at the same time of day but across three different days, and on the same physical bus. The bus had one door for entrance and one for exit.

**WLAN probe data:** these data types consist of WLAN probes, passively collected from a sensor placed on the bus under the roof, approximately in the center of the bus. The WLAN probes are emitted by WLAN-enabled devices in and outside the bus. For each WLAN probe collected, the time stamp and GPS location is recorded along with the recorded signal strength of the received probe.
**Light sensor data:** these data types consist of enter and exit events recorded by light sensors placed in the entrance and exit of the bus. The events are recorded per bus stop on the bus route.
**Ground truth data:** these data types consist of enter and exit events manually counted by a person per door riding in the bus. The events are recorded per bus stop on the bus route.
**Data set usages:**Table 1 presents an overview of what data types are available in which data sets.

DS1 and DS2 are used to calculate error probabilities of the light sensor estimator. DS3 and DS4 are used to determine parameters for the WLAN probe-based estimator, and are used in the final comparison. DS5 and DS6 are used for the comparison of the light sensor estimator and the WLAN probe-based estimator. DS5 and DS6 are also used to assess the fusion approach.

Data sets collected on the same day were obtained from bus trips on the same bus route, but in different directions. Furthermore, they were recorded immediately after each other.

The choice of WLAN probe sensor location was influenced by the bus model and by the need for a local power connection. The used bus model, Mercedes-Benz Citaro O530N, with two doors, only provides a few mounting locations, which also offer a power source. One location being in the front of the bus next to the driver and one being under the roof near the center of the bus. To keep deployment costs minimal, only a single WLAN probe sensor was used for one bus. In any case, as the WLAN transmission range easily covered the whole bus, multiple sensors would have had little effect, unless they used directional antennas. Processing of probe data from multiple sensors with directional antennas can help to identify devices that are on the bus; however, the effort to correctly mount and calibrate these sensors would increase drastically.

## 4. WLAN Probe Based Estimator

In this section, the estimator based on WLAN probes is described. This includes describing the WLAN probe processing methodology and converting the estimated number of devices to a number of people.

### 4.1. WLAN Probe Collection System

**Figure 1:**
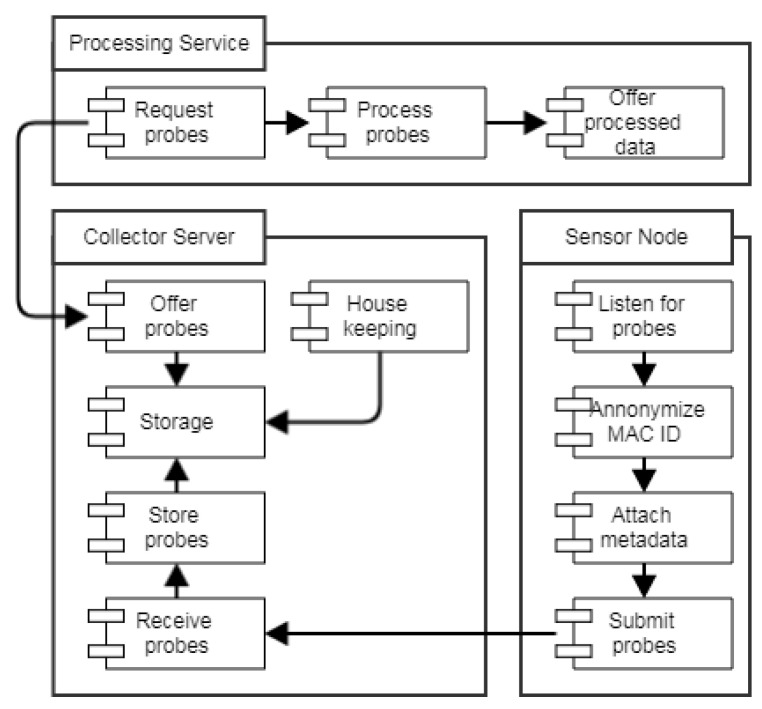
WLAN probe collection system architecture: the sensor node (lower right) is placed on the bus and it receives probes from WLAN devices. The collected probes are, after some processing, sent to the collector service (lower left), which stores them and makes them available to services for further processing. The processing service (top) retrieves the stored probes and processes them, in the case of this paper, for estimation of the bus occupancy.

Figure 1 presents the architecture of the system for collecting WLAN probes from WLAN-enabled devices on buses. WLAN probes were collected passively, meaning no connection was established between the sensor and the devices emitting the probes. This was possible because the probes were broadcast by the devices as a first step in querying if the known access points were near, and if so, to connect with them. The broadcast scheme does however also mean that the sensor might not capture all probes due to collisions with probes from other devices. Furthermore, due to the device emitting the probes deciding the frequency with which to emit the probes, there is no certainty that probes from all devices will be captured within a given time interval.

A sensor node is placed on a bus where it collects WLAN probes from devices, attaches the GPS location and time stamp, and pushes the data to the collector server. The collector server stores the probes and offers them to various processing services, which utilize the probes for purposes, such as estimating the live bus location, live bus occupancy, and other. The processed data are finally offered to other services or applications that might need it.

The data flow in the architecture is designed and developed to be a demonstration use case of the BIG IoT project [21], where data and services are offered, using a common BIG IoT API. The goal of the BIG IoT project is to enable interoperability among IoT platforms, services, and applications by streamlining interfaces. Offering data and services via a streamlined interface means that services and applications consuming the offerings only have to conform to a single interface. This makes the entry barrier of obtaining data extremely low, and usability of service APIs high. The BIG IoT API defines how to describe data offerings, and provides methods for discovering offerings via a number of different filtering options. Furthermore, the API also provides data access control, further minimizing the effort of offering, providing and consuming data among services and applications.

### 4.2. Processing of WLAN Probes

The WLAN probe-based estimator (henceforth named WLAN estimator) earlier described in [3] is an approach to estimate the number of devices on the bus based on applying threshold filtering to the WLAN probes. There are two threshold values; one for RSSI value and one for device presence time. The RSSI threshold is applied to filter away probes with too low RSSI values. The assumption is that low RSSI probes the origin from devices far away from the sensor, i.e., outside the bus. After the probes are filtered based on the RSSI value, the time threshold is applied. This threshold is evaluating the duration between the first probe from a device that satisfies the RSSI threshold and the last. If this duration is above the time threshold, the device is evaluated as being on the bus. This is based on the assumption that devices traveling on the bus will be seen for a duration, which is longer than devices outside of the bus passing by.

**Figure 2:**
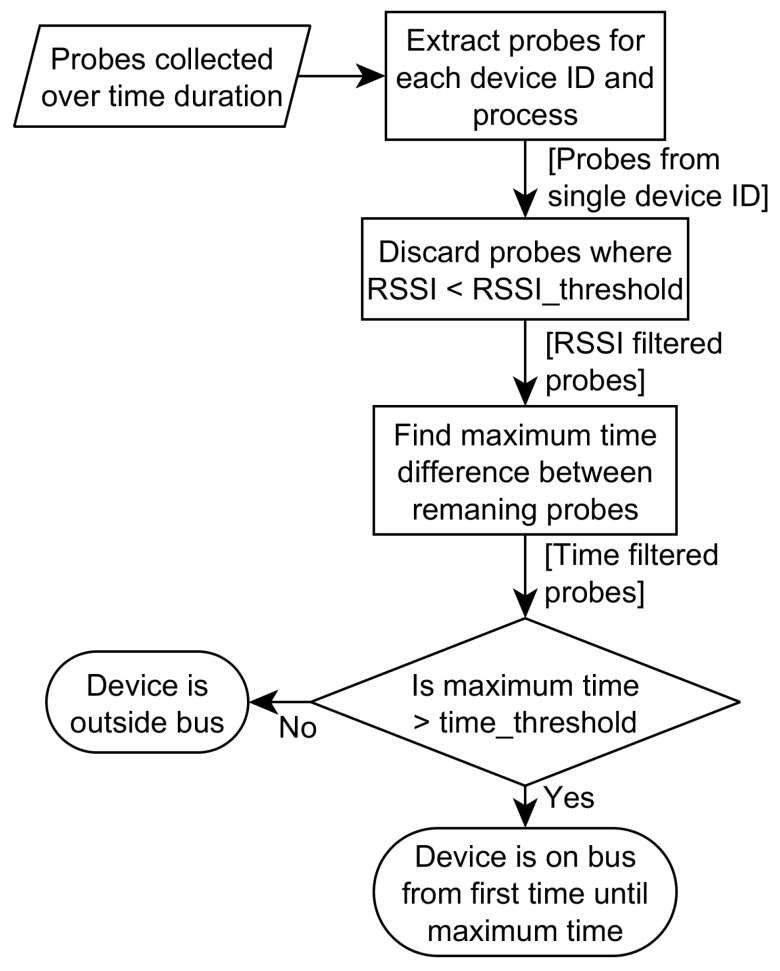
Flow of the WLAN estimator algorithm showing how WLAN probes are processed by applying the two-threshold filtering in order to decide whether the device is on the bus or not.

In Figure 2, the flow of the WLAN estimator algorithm is illustrated. Based on a set of probes collected over a time interval, the algorithm applies the two threshold filtering steps on a subset of probes per device ID and decides if that device is inside or outside the bus.

### 4.3. Number of People Based on Estimated Devices

The WLAN estimator estimates the number of devices; to get the number of people, an extrapolation from the number of devices carried per person is required. We will initially use an extrapolation based on statistical data, which are described in this subsection. Later, in Section 9, a novel approach based on fusion of outputs from WLAN and LSE will be presented.

The statistical approach is also presented and described in [22]. Based on numbers from Statista, the number of smartphone users in Germany in 2017 was 55.46 million [23], and the population in 2017 was 82.65 million [24]. These numbers give a penetration of 67 percent of the population with a smartphone, or 0.67 smartphones per person.

In the data collection process for this paper, the actual number of devices per person is also derived based on polling of travelers on the measured bus. This is done based on interviews of passengers during the two bus trips corresponding to DS3 and DS4, where the persons entering the bus are asked how many smartphones they carry. On these 2 bus trips, a total of 86 persons entered the bus, and a total of 54 smartphones were counted. From this, it was found that the expected number of devices per person was 0.63, which is close to the estimated number of smartphones per person based on the Statista numbers [23,24]. Except for Section 9, the subsequent results use the number of 0.63 devices per person.

This factor will be applied to the WLAN estimate for the number of devices, as indicated in Equation (1).

(1) $$Estimated_{people}=\frac{Estimated_{devices}}{0.63}$$

The probability that a person is a smartphone user or not, does not consider whether a person carries more than one smartphone or other WLAN-enabled devices. To get a full picture of the number of WLAN-enabled devices per person, a demographic analysis would be required, taking into account different tendencies for different demographics. In this case, a distribution should be derived, indicating the probability of a person carrying 0, 1, 2, etc., devices. Estimated distributions for that have been used in [4]; however, due to the lack of sufficient empiric data, this work only uses the mean value and, hence, applies Equation (1).

## 5. Alignment of WLAN Estimator with Bus Stops

In this work, the WLAN estimator is compared with a light sensor-based system, which only registers changes at bus stops activated by the door opening. As the comparison metric is calculated just after each bus stop, the WLAN estimator needs to be aligned to the time instants, when the bus departs from a bus stop. However, the WLAN sensor has no knowledge about the bus stops; hence, this alignment requires the correlation with another data set. For this purpose, the locations of the bus stops are used, which are matched with the location of the WLAN probe sensor.

The WLAN estimator estimates the number of people on the bus, independent of bus stops, where people actually enter and exit the bus. Here, "people presence" is based on the reception of WLAN probes, where the first probe can be received before or after the person carrying the device physically enters the bus, and the last probe can be received either before or after the person exits the bus, due to the transmission range of WLAN. This means that a device registered as being inside the bus can be shifted in time compared to when it actually is inside the bus, and similar for when the device exits the bus.

It is assumed that the RSSI filtering applied in the WLAN estimator removes most of the probes received from devices while they are outside the bus. This means that the first probe is often received after the device enters the bus, and the last probe is received before the device exits the bus. This leaves the device presence as being shifted in time, between the first and last bus stops for the device in question.

Based on this, the WLAN estimator is aligned by pushing exit events to the next bus stop after they are registered, and enter events are pulled to the bus stop immediately before they are registered. This approach will also ensure that the estimated number of people on the bus does not change while the bus is driving. Note that, depending on the probe emission frequency and the time between bus stops, the alignment algorithm presented here may not achieve the correct alignment, if there were additional bus stops between the first/last probe and the actual entering/departure bus stop.

### 5.1. Alignment Approach

The alignment is done by comparing GPS locations of bus stops with GPS locations of the collected WLAN probes. For this reason, the geographical locations of the bus stops are used and obtained from the bus operator’s database. Note that the bus stops are typically at fixed locations. Small deviations of several meters are considered in the proposed scheme. Large deviations of bus stop locations, e.g., due to construction sites, are not addressed in the scope of this paper. It is also assumed that the WLAN probes are sorted chronologically, i.e., the time stamps are non-decreasing, and that the bus stops are listed in the order that they are visited by the bus.

The first bus stop is matched by identifying the GPS coordinate of the WLAN probe closest to the bus stop, notably within 20 m. Furthermore, it is also checked that the speed is sufficiently low (below 1 m/s), i.e., the bus is slowing down. The obtained location and time stamp from the WLAN probe data are marked as the bus stop. This means that the marked time and location is when the bus arrives at the bus stop. The alignment approach is described in the following pseudo code:

1. For each bus stop, get the location $\left\{lat_{bs},lon_{bs}\right\}$.
2. Select the subset of probes with $dist(\left\{lat_{bs},lon_{bs}\right\},\left\{lat_{probe},lon_{probe}\right\})<20$ m.
3. Calculate the speed for the subset of probes (geographical distance between two consecutive probes divided by the time interval length between the same probes).
4. Select the first probe where speed is below 1 m/s.
5. Mark the time and location of the selected probe as the start of the bus stop.

This procedure is performed repetitively for the remaining bus stops on the route, until the WLAN probe data are exhausted or the last bus stop is reached.

An exception might occur in the case the bus passes by the bus stop without stopping, i.e., no passengers get on or off. These bus stops are still registered in the WLAN probe data in a second iteration of the processing. Here, the location and time stamp of the bus is marked when the distance between the bus and the bus stop is smallest. The change of bus occupancy for the passed bus stop is naturally 0.

The WLAN estimator outputs the start and end times of when a device ID is visible. This is utilized in the alignment, by going through all device IDs and changing the start time to that of the bus stop before the start time, and changing the end time to that of the next bus stop after the end time.

An issue might occur in the scenario where the bus stops at a traffic light or in a traffic jam within 20 m of the bus stop, before stopping at the bus stop moments later. In this case, the bus stop will be registered as the location the bus stops at the traffic light. This means that the time stamp assigned for the bus stop will be wrong, and the entering and exiting events will be registered earlier than the actual bus stop. However, for the purpose of comparing the estimated bus occupancy from two estimators at bus stops, this does not have a critical influence on the results, so it will be tolerated for now. The low speed of a bus outside a 20 m range from a bus stop, either due to a traffic light or a traffic jam, will not be registered as a bus stop.

### 5.2. Evaluation of Alignment

**Figure 3:**
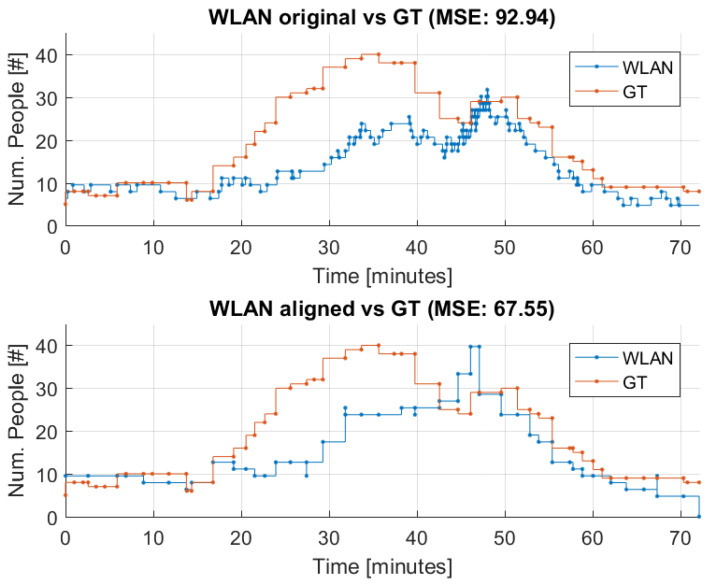
Comparison of WLAN estimator and GT before and after the alignment of the WLAN estimator based on dataset 3 (DS3).

In Figure 3, the WLAN estimator is compared to ground truth (GT), both before and after the alignment. On this figure, the bus occupancy is indicated over time to illustrate how the enter and exit events are accumulated.

To evaluate the impact of the alignment of the enter and exit events of the WLAN estimate, the difference, or error, between GT and the WLAN estimator is evaluated. This is done by calculating the mean squared error (MSE) at each bus stop where data are available for both the WLAN estimator and GT. MSE is calculated according to Equation (2).

(2) $$MSE=\frac{1}{B}\sum_{i=0}^{B}{\left(GT_{i}-WLAN_{i}\right)}^{2}$$

where *B* is the number of bus stops, $GT_{i}$ is the ground truth bus occupancy, and $WLAN_{i}$ is the WLAN probe estimate.

The average MSE for the original and the aligned WLAN estimator are listed in Table 2, along with the improvement from the alignment in the percentage. The MSE is calculated for a set of eight representative threshold value pairs (listed in Table 3).

From Table 2, it can be concluded that the aligned WLAN estimator is closer to GT than the original WLAN estimator, and the improvement justifies to apply this alignment approach. Henceforth, the WLAN estimator results presented will be aligned according to bus stops.

## 6. Selection of Parameters for WLAN Probe Based Estimator

As described earlier, the WLAN estimator applies threshold-based filtering to estimate if devices are on or outside the bus. For this reason, the selection of these threshold values will impact results of the estimator. The threshold values are related to minimum travel times of devices, and to the signal strengths of collected probes. This means that the choices of threshold values depend on the placements of the sensors on the bus, and on the type of routes the buses drives, i.e., if there is a long or short time between bus stops. For this reason, the threshold values must be chosen, based on the specific scenario that the system is deployed in. In the specific scenario considered in this paper, the time between consecutive bus stops is between 1 and 3 min. Based on this, values for the device presence time threshold are chosen between 30 and 180 s.

**Figure 4:**
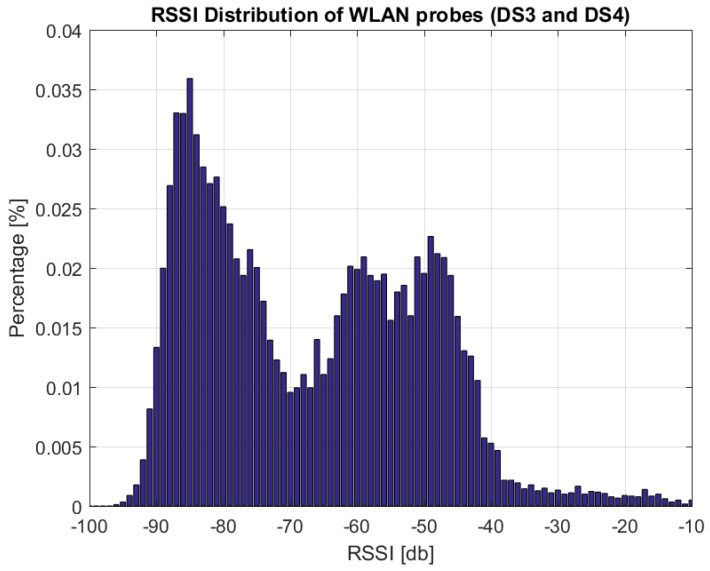
Distributionof registered RSSI values of collected WLAN probes in datasets 3 and 4 (DS3 and DS4) (bin size 1 dB).

In Figure 4, the distribution of RSSI values recorded for the collected WLAN probes in DS3 and DS4 is presented. The distribution reveals two main parts, namely a set of RSSI values from −95 to −70 dB and a second set from −65 to −40 dB. Taking the assumption that the lower set of RSSI values originates mainly from devices outside the bus, and that the larger set of RSSI values originates from devices placed in the bus, a threshold approach can split these two value regimes. Based on the shape of the histogram in Figure 4, thresholds between −80 and −60 db will be analyzed for that purpose.

Based on these two ranges, all combinations of {−80dB:2dB:−60dB} and {30s:30s:180s} are evaluated. For each of the threshold pairs, the estimator is applied to the data. Then the error is evaluated between the estimate and the GT by calculating the mean squared error (MSE) at each of the bus stops. The threshold pair to be used is selected by calculating the average MSE per threshold pair over the used data sets (in this case, DS3 and DS4), and selecting the threshold pair yielding the smallest average value, i.e., $MSE=(MSE_{DS3}+MSE_{DS4})/2$.

In Table 3, the eight threshold pairs with lowest average MSE values are shown. Based on this, threshold pair 2, i.e., −76 dB and 120 s, is chosen. Note that the fine tuning of parameters and choosing a good pair from the plausible set was done using the knowledge of the ground truth. As GT measurements can not be known for all bus rides, we keep this set of parameters and use them for the WLAN-based estimator, and when other days are considered. In Section 8, this set of threshold values is used for DS5 and DS6, even though it might not be the most optimal one.

**Table 3:** Evaluation of threshold pairs via MSE between GT and WLAN estimator.

Id	Threshold Pair	MSE DS3	MSE DS4	Avg. MSE
1	{−78, 120}	60.43	29.21	44.82
2	{−76, 120}	61.48	21.23	41.36
3	{−76, 30}	62.88	23.01	42.95
4	{−76, 60}	61.39	25.55	43.47
5	{−76, 150}	67.73	21.95	44.84
6	{−74, 30}	63.33	20.12	41.72
7	{−74, 120}	67.49	21.33	44.41
8	{−72, 30}	67.55	21.22	44.38

## 7. Light Sensor Based Estimator

In this section, the light sensor estimator (LSE) is presented, along with comparison with ground truth measurements to obtain error probabilities of the estimator.

### 7.1. Light Sensor System

The light sensor-based passenger counting system, installed in 22 buses of Wolfsburg Verkehrsgesellschaft mbH, is based on the DILAX system [25]. The sensor system consists of highly sensitive active infrared detectors. They permanently emit light pulses that are reflected by passing passengers and registered by the system. Passengers only use the front door for entrance and the back door for exit, so the sensor detects the movement on each specific door when the door is open. The light sensors are placed on top of the front or back door area. Sources of errors identified can be passengers carrying bigger objects, such as bikes, prams, or wheelchairs, but also bigger suitcases. Note that, depending on the specific use-case, the detection of large objects by the light sensor can be a desired result. However, here, the focus is on passenger numbers in the evaluation.

The results are collected at a so-called people-counting unit. From the vehicle, the collected data are transmitted via a Wi-Fi connection to the DILAX-server. This happens when the bus returns to the garage. A software monitoring system allows evaluating the data captured in the bus. The measurements are verified, processed, and grouped in different reports or indicators: passenger numbers are analyzed per line, trip, stop, or direction, as well as type of day, period, or others.

### 7.2. Light Sensor Estimator (LSE) Approach

For each bus stop in the light sensor data, the number of people entering or exiting the bus is registered in an onboard unit. These events are stored as instantaneous events, meaning that a single scalar value for people entering and a single scalar value for people exiting at each bus stop. From this, the number of people on the bus over time is obtained by cumulatively adding the number of people entering and subtracting the number of people leaving. This however means that if there is an error in counting, this error will remain in the estimated number of people until the count is reset. The reset of the counting is done in the post-processing of the data, i.e., based on the assumption that the bus is empty at the start of a bus trip. This can result in the number of people becoming negative. This error will be evaluated in the following section, by evaluating enter and exit events separately.

### 7.3. Evaluation of Error Probability

As stated previously, the light sensors do not register all enter and exit events. To investigate this error, the events are compared to the ground truth, obtained from two bus trips with 51 and 52 bus stops, respectively (data sets DS1 and DS2).

**Figure 5:**
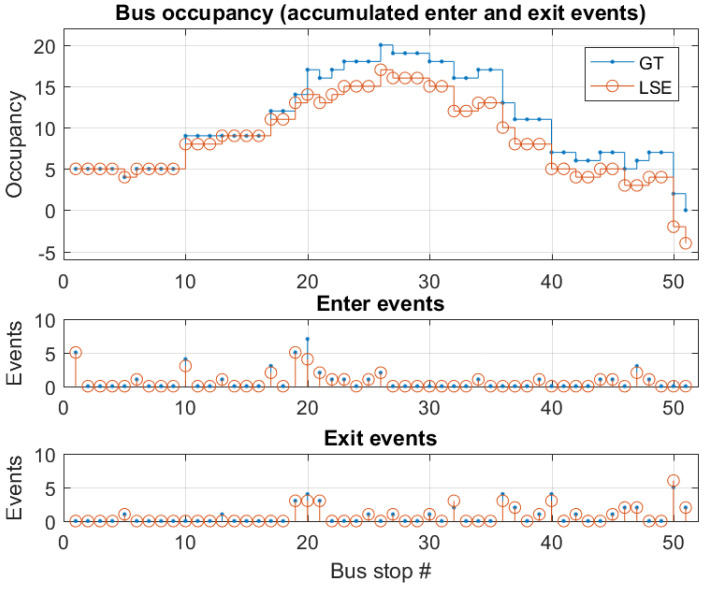
Top: Busoccupancy GT and LSE per bus stop. Middle: enter events per bus stop. Bottom: exit events per bus stop. Based on dataset 2 (DS2).

The error is evaluated for enter and exit events separately, by calculating the probability of different types and scales of errors. Next, the enter and exit events are combined to probabilities of the LSE deviating from GT. In Figure 5, GT and LSE data collected on bus trip DS2 is presented, along with indication of the number of enter and exit events per bus stop.

**Figure 6:**
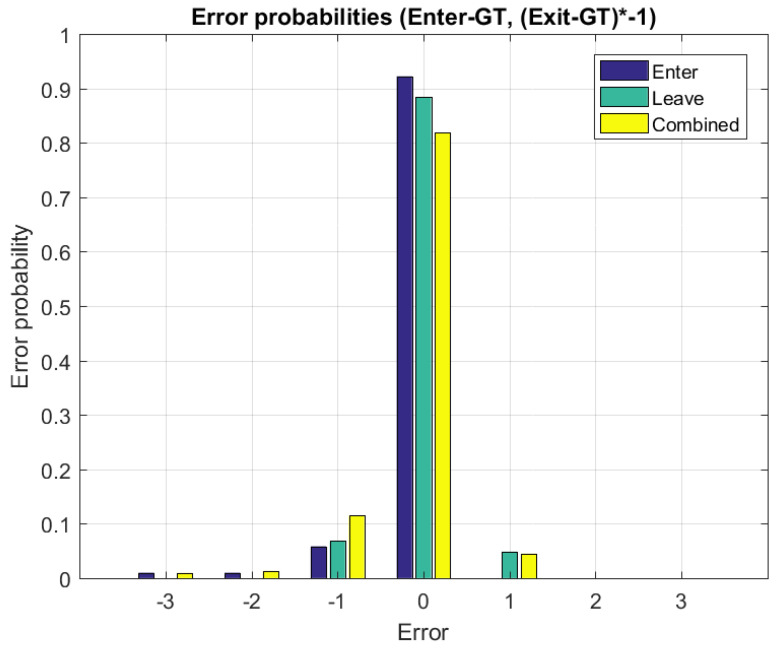
Probabilities of enter and exit event errors, and of combined events. Note: in order to combine enter and exit event errors, exit events must be multiplied with −1 before calculating probabilities, as exit events are people leaving the bus. The expected combined error: −0.1237. The empiric distribution was obtained from data sets 1 and 2 (DS1 and DS2).

The empiric distribution of the differences of the LSE enter events and of the GT enter events, and of the differences of the GT exit events and the LSE exit events (note the inverse sign) for the bus trips DS1 and DS2 are shown in Figure 6. The convolution of these two distributions, or analog, the empiric distribution of the LSE occupancy change per bus stop minus the GT occupancy change per bus stop, is also shown in Figure 6. The latter shows a mean error per bus stop of −0.1237, i.e., the LSE shows some drift to smaller occupancy values as compared to the GT bus occupancy in these two bus trips. This is also seen in the right end of Figure 5, where the LSE becomes negative, while GT becomes 0.

**Figure 7:**
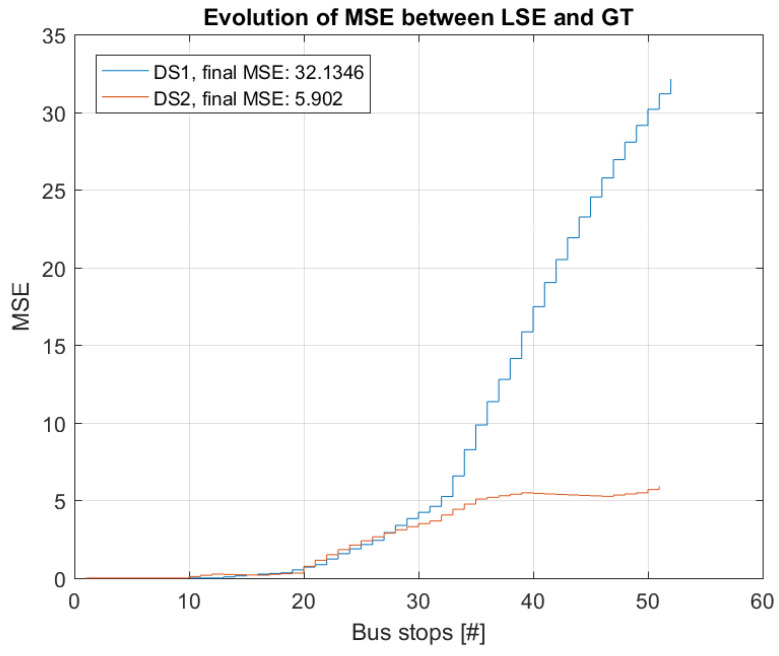
Evolution of MSE between LSE estimated occupancy and GT occupancy for two bus trips. Results are shown for the bus trips in data sets 1 and 2 (DS1 and DS2), respectively.

To illustrate the accumulating error of the LSE estimator, the MSE is calculated between GT and LSE occupancy for an increasing number of bus stops along the bus route. In Figure 7, the evolution of the MSE is presented for DS1 and DS2. From this, it is apparent that the error accumulates, i.e., the MSE shows a trend to increase over an increasing number of bus stops in the route for both data sets.

## 8. Comparing Estimators

To evaluate how the two estimators perform compared to each other, data sets DS5 and DS6 are used. Note that these data sets are different from data sets used for calibration of the WLAN estimator (DS1 and DS2) and for the error analysis of the LSE (DS3 and DS4). This comparison is done based on different approaches. First, the estimated bus occupancy is plotted with the ground truth to get a general understanding of how close the estimates are to the ground truth. Next, the MSE is calculated per estimator, per bus stop, to evaluate the error between the ground truth and the two estimators, respectively. The evolution of the accumulated MSE is plotted to understand how the error develops for the two estimators.

**Figure 8:**
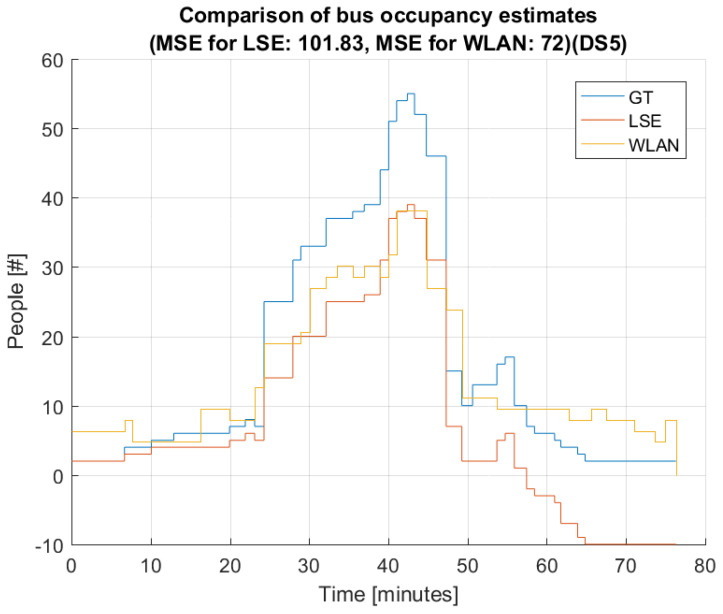
Comparison of LSE and WLAN with GT based on data set 5 (DS5).

**Figure 9:**
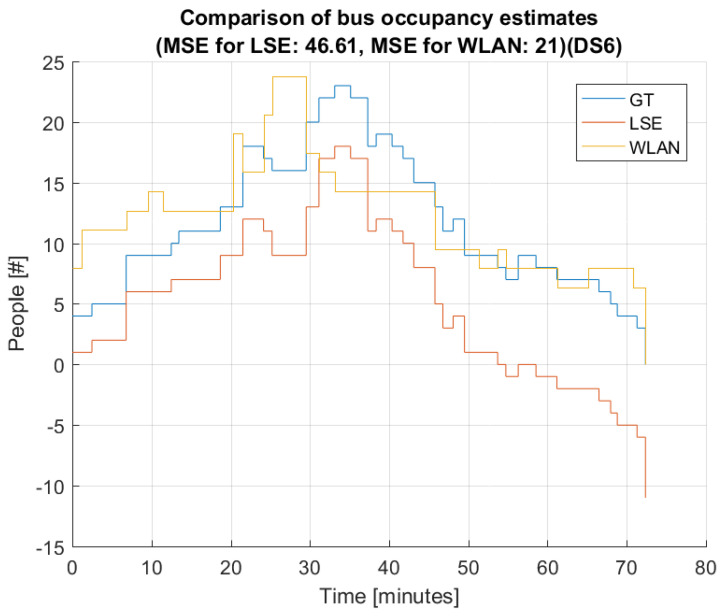
Comparison of LSE and WLAN with GT based on data set 6 (DS6).

Figure 8 and Figure 9 present the estimated bus occupancy and GT based on DS5 and DS6, respectively. From these plots, it is seen that both estimators generally follow the GT. For DS5, the WLAN estimator generally follows the shape of GT, but results in both over- and underestimations. The LSE estimator mostly follows changes in GT; however, over time, small errors accumulate that it never recovers from. This means that the LSE estimates a negative value in the last part of the bus trip.

For DS6, the WLAN estimator again generally follows the shape of GT throughput the bus trip. However, at around 24 minutes, the WLAN estimator sees a huge spike, which is not present in the GT. This might be due to the bus being stopped at a location where the amount of people outside the bus is high. The LSE estimator behaves similarly as for DS5, in that it generally follows the shape of GT, but with small errors here and there, which accumulate. This means that, again, in the last part of the bus trip, LSE estimates a negative number of people on the bus.

**Figure 10:**
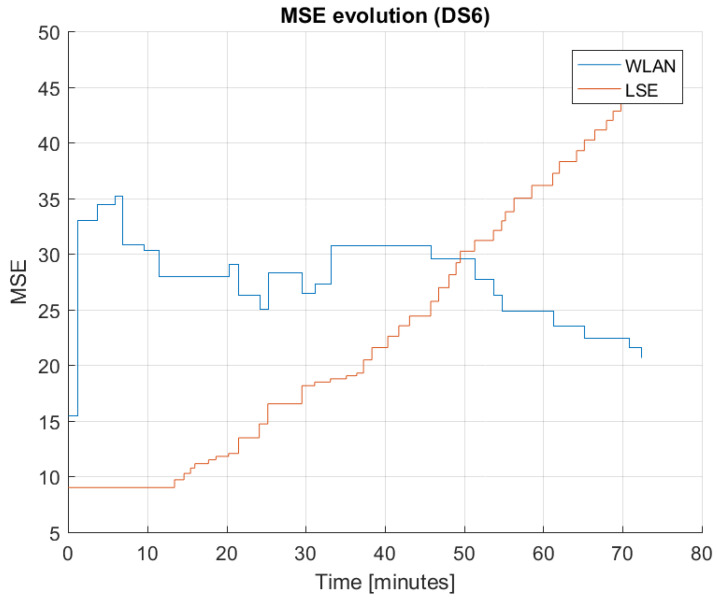
Evolution of MSE between GT and LSE or WLAN based on data set 6 (DS6).

It is important to observe how the error evolves with time, as this gives good insight into estimator behavior. In Figure 10, the evolution of MSE is plotted for LSE and WLAN for DS6, and this figure presents a typical behavior of estimation errors over time, which is in correspondence with the estimator nature. The LSE MSE increases from start to end, with almost a linear increase throughout the bus trip. The WLAN MSE fluctuates in an interval between 25 and 35, being lower at the beginning and at the end of the trip.

The observation regarding typical behaviour of MSEs of different estimation methods will be used for improved estimation in the next section.

## 9. Improved Estimation by Fusion of WLAN and LSE information

We saw in the previous section that both WLAN and LSE estimators show some substantial estimation error. It is possible to make further improvements on the estimation accuracy. In [22], the WLAN probe-based estimator is improved by modeling false positives and false negatives, meaning devices wrongly estimated as being either on the bus or outside the bus; however, this approach requires some additional measurements.

Knowledge of ground truth can be used to set up the estimation algorithm parameters in an optimal way. In Section 6, a pair of threshold values is selected to minimized average MSE over different rides. Additionally, in [4], the derived empiric error distribution for the LSE estimator is used to calculate confidence intervals and to eliminate the bias of the LSE estimator. These methods yield estimation improvement, but require GT knowledge. Obtaining GT is a manual and costly process, which is not scalable. Since parameter adjustment should be done individually for different operating conditions (bus type, typical passenger load, area characteristic), it is not realistic that massive manual calibration of the estimators can be done in practice.

Instead, we would like to propose a new approach for estimation improvement, based on fusion of data coming from both WLAN and LSE estimators. It can be more correct to call this approach *information fusion*, as we are working with already processed data and not with raw data collected by the sensors.

### 9.1. Information Fusion Approach

For estimation of the number of people on a bus, we introduce a correction part that is modeled based on the understanding of the physical nature of the data collection process and errors occurring during this process. In case of the WLAN estimator, the correction has a form of a multiplicative factor, and in case of the LSE estimator, it is an additive correction term. This modeling approach is also supported by the measurement data presented in Section 8 (see Figure 10). Since the behavior of MSEs is different for two estimators, knowing both of them allows us to calculate the correction factors.

The proposed approach can be used in cases when the WLAN probe measurement sensor and light sensor are installed in a bus. In case only a single measurement system is in use, it can be temporally supplemented with another system to find a value for the correction factor/correction term. Subsequently, the found correction part can be applying to the system in use, as long as there is no essential changes in the operating conditions. This will provide an improved estimator and remove the need for laborious manual people counting.

### 9.2. Parametric Modeling

Here, we describe the mathematical modeling proposed for finding correction parts. Let $W_{i}$ be the estimator of the number of people obtained from the WLAN device counting between bus stop *i* and i+1; that is, $W_{i}$ is the output of the WLAN estimator that can be obtained, as described in the previous sections. We assume that bus occupancy $O_{i}$ can be obtained by a multiplicative model:

(3) $$O_{i}=c_{i}\cdot W_{i},$$

in which the random variable $c_{i}$ is introduced to compensate for device estimation errors and for errors connected with mapping from the number of devices to the number of people. The following assumption is made about distribution of a r.v. $c_{i}$: $c_{i}$ is assumed to have an expected value that is independent of the time interval *i*,

(4) $$\omega :=E(c_{i}).$$

Equation (3) can be rewritten as:

(5) $$O_{i}=\omega \cdot W_{i},$$

In order words, a multiplicative error factor for WLAN estimator is assumed to be constant for all intervals between the bus stops.

The model for the LSE assumes an additive error $\epsilon_{i}$, as follows:

(6) $$O_{i}=\sum_{j=1}^{i}\left(E_{j}-L_{j}+\epsilon_{j}\right),$$

where $E_{i}$ and $L_{i}$ are the estimated number of enter, respectively, and leave events at bus stop *i* from the LSE. We assume here also that the expected value of $\epsilon_{i}$ is independent of *i*:

(7) $$\lambda :=E(\epsilon_{i}).$$

Similar to an assumption that a multiplicative factor, to compensate for an error in WLAN estimator, is a constant, we conducted a similar assumption of a constant additive error factor to an LSE estimator that does not depend on the time/bus stop number. Assuming that there are *K* bus stops, and since the right hand sides of Equations (3) and (6) are the same, we have the following *K* equations:

(8) $$\omega \cdot W_{i}=\sum_{j=1}^{i}\left(E_{j}-L_{j}+\lambda \right),$$

which can be rewritten as

(9) $$\left[\begin{array}{cc} W_{1} & -1\\ W_{2} & -2\\ \ldots \\ W_{k} & -k \end{array}\right]\cdot \left[\begin{array}{c} \omega \\ \lambda \end{array}\right]=\left[\begin{array}{c} E_{1}-L_{1}\\ E_{2}-L_{2}+E_{1}-L_{1}\\ \ldots \\ E_{k}-L_{k}+\ldots E_{1}-L_{1} \end{array}\right].$$

Equation (9) is solved for the minimum MSE and, thus, the estimations for correction parts ω and λ are obtained.

### 9.3. Results

We now show the improvements from the estimation of ω and λ via the previously described approach on the traces DS5 and DS6. Since this approach is applied in situations when the ground truth is not available, the optimization of the parameter choices for the WLAN estimator, as done in Section 6, is not possible. This is why we chose to illustrate the information fusion approach with a randomly selected pair of threshold parameters for the WLAN estimator. In the following, an RSSI threshold of −75 dB and a time threshold of 10 s are used. Note that the suggested approach will work on any pair of the threshold parameters.

**Figure 11:**
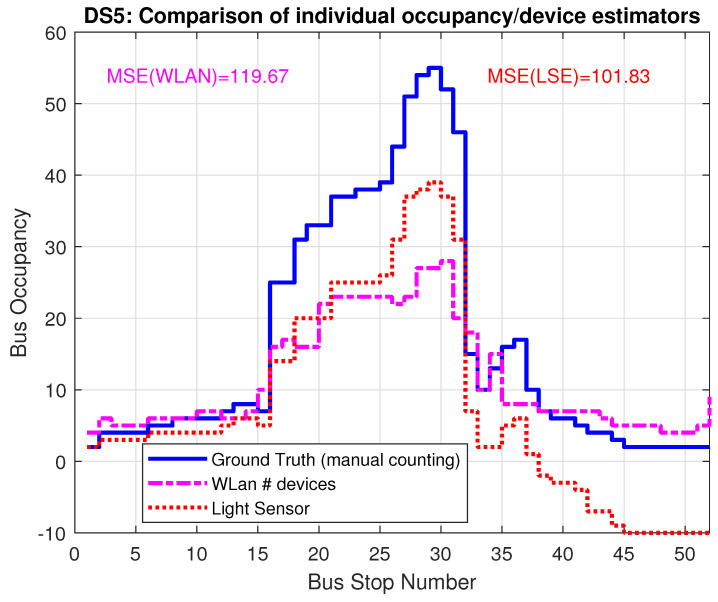
Bus occupancy (LSE) and number of detected devices (WLAN) for trace DS5 WITHOUT inclusion of λ and ω from the fusion approach.

Figure 11 shows, as the starting point, the estimation when applying the WLAN estimator and the LSE individually for data set DS5. As shown earlier, the LSE underestimates the occupancy, which ultimately leads to even negative estimates to the right end of the figure. The MSE of both estimators is above 100.

**Figure 12:**
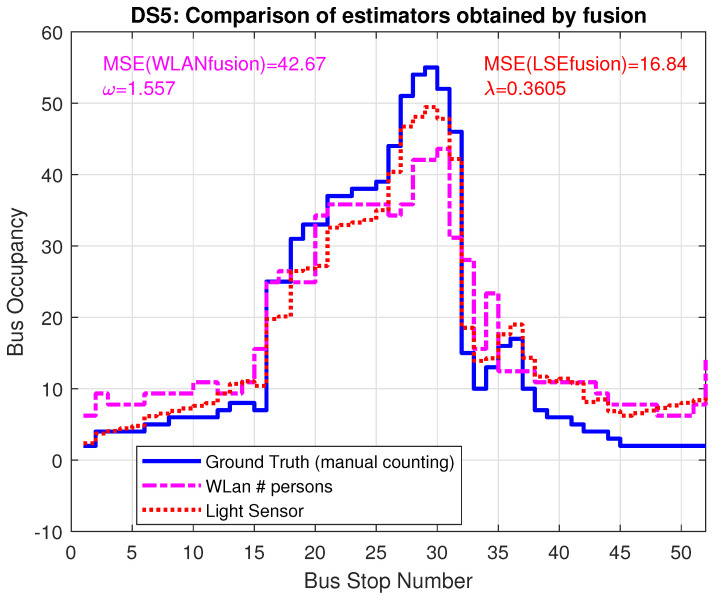
Bus occupancy for corrected models LSE and WLAN for trace DS5, including λ and ω from the fusion approach.

After calculating the correction parts, the factor ω=1.557 for WLAN and the additive term λ=0.385 for LSE, Figure 12 shows the improved estimators, resulting from the combination of LSE and WLAN data. The MSE improves significantly, by a factor of 2.8 for WLAN and a factor of 6 for LSE and the curves show a much better match to ground truth.

**Figure 13:**
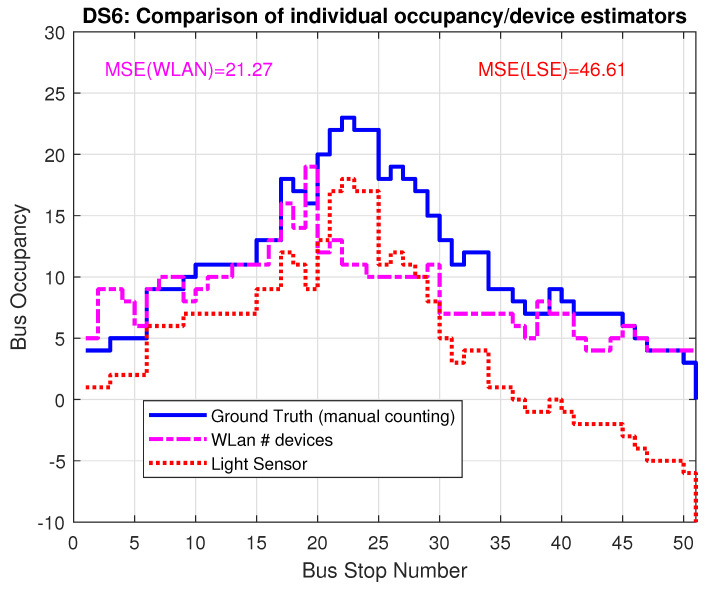
Bus occupancy (LSE) and number of detected devices (WLAN) for trace DS6 WITHOUT inclusion of λ and ω from the fusion approach.

**Figure 14:**
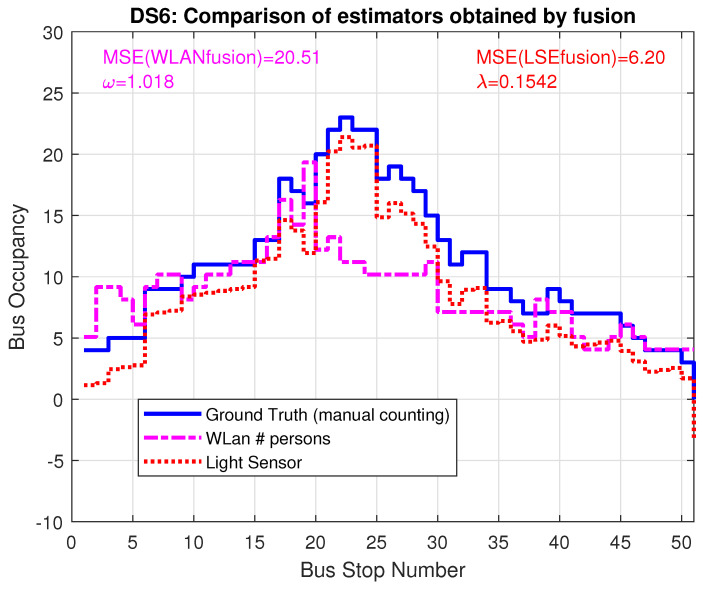
Bus occupancy for corrected models LSE and WLAN for trace DS6, including λ and ω from the fusion approach.

The improvement for trace DS6 is shown in Figure 13 and Figure 14: for this data set, the WLAN estimate was already quite good without any correction factor; the resulting γ=1.018 therefore only provides minor improvement. The LSE, on the other hand, improved by a factor of 7.5 to the resulting MSE = 6.2.

Compared with the optimization of the WLAN estimator parameters utilizing GT knowledge, the fusion approach yields even better performance: for trace DS5, the clever threshold pair selection results in MSE equal to 72; while fusion approach reduces MSE to 42.67. For DS6 the difference is not so drastic: threshold selection brings MSE down to 21; while the fusion approaches reduces it to 20.51.

Results show that the joint processing of WLAN and LSE data yield corrective terms, which can improve both estimators. The results of DS6 however show that it is advisable to use the corrected LSE, while the corrective additive term can be calculated from inclusion of the WLAN data without any knowledge of ground truth.

## 10. Conclusions and Discussion

In this paper, two passive passenger count estimation approaches were compared. This was done based on data collected through the two systems in a live deployment in buses in a medium-sized German city. The two methods were chosen to represent, on one side, the established approach in form of the light sensor-based system, and on the other side, the more experimental approach in form of the WLAN probe-based approach. Both systems are non-intrusive, in that they do not require any action from passengers.

The main difference between the two approaches is that the light sensors directly sense passengers passing through the doors, while the WLAN probes indicate devices in the vicinity, which must be used to extrapolate the number of people. The comparison is done by comparing the error and the evolution of error between the estimated number of passengers and the ground truth, where the error is calculated, in terms of the mean squared error. These parameters were chosen because they indicate the accuracy of the estimator during operation. GT is obtained by manual people counting during bus rides.

Based on the comparison of the two estimators with the GT, a number of conclusions can be drawn. Generally, both WLAN and LSE follow GT, in terms of overall bus occupancy characteristics. The WLAN estimator experiences both over- and underestimation, but generally follows the shape of the GT. LSE is able to follow the fluctuations of GT in greater detail, but the small errors that occur are accumulating. While LSE is not able to recover from these errors, the WLAN estimator is able to recover, even after larger errors.

Another thing to consider is the delay from when the bus occupancy changes due to passengers getting on or off the bus, until when the information is available based on the estimation system. In the WLAN system, the delay is caused by the chosen time threshold and by the data transfer schedule and speed from the sensor to the collector server. For the light sensor, the delay is smaller, i.e., only depending on the data transfer schedule and speed from the sensor system on the bus to the back end.

It should be noted that the results for LSE presented in this work only apply to the current setup, i.e., a bus with a door for entrance and a door for exit. If doors are multi-purpose, then either the sensor should be able to distinguish the event type, or the data should be processed in another way. The results presented for WLAN are independent on how bus doors are used during operation. However, the approach would have to be recalibrated in terms of threshold value selection. Independent of the specific physical setup—the WLAN estimation needs to extrapolate from the number of devices to the number of persons, and this extrapolation step adds some uncertainty to the estimator.

From this, it is concluded that there are advantages and disadvantages with both approaches. LSE is better in following GT in detail, at a short-term, while suffering in the long-term. Conversely, the WLAN estimator is not good at following GT in detail at a short-term, but at a long-term it follows the general tendencies of GT. Regarding live deployment, both the LSE and WLAN estimators are viable approaches for estimating bus occupancy, but with different strengths and weaknesses for each estimator.

The comparison of the estimators reveals that there is room for improvement in both estimators. We proposed a novel approach, improving the accuracy of the estimations based on information fusion. The core observation applied in the fusion is that the error behavior of the two estimators is very different. Different types of errors in data sources allow for error compensation in the fusion process. A multiplicative model for error compensation is used in the WLAN estimator, while for the LSE estimator, the error compensation is introduced as an additive term. Having measurement data from both estimators allows calculation of the correction factors. Results show a significant improvement in MSE and the achieved MSE is minimal compared to other approaches that we applied for parameter fine-tuning.

The clear advantage of the fusion approach is that this optimization method does not require knowledge of ground truth, which is necessary for fine-tuning the WLAN estimator to achieve good accuracy. Ground truth can be obtained by manual counting, and it is a very costly and laborious process, which is also error-prone. Using another sensor-based measurement system, temporally or permanently, for estimation correction, can provide necessary scalability and automatism.

Additionally, the WLAN probe-based estimation requires extrapolation from the number of devices to the number of people. Such extrapolation introduces additional errors, as it relies on a prediction of how many devices people are carrying. The correction factor calculated in the fusion approach automatically accounts for this mapping and can provide a necessary factor without any use of demographic statistics or similar information on distribution of mobile devices within the population.

The results presented in this paper are based on real-life measurements; this serves as a good indication of what performance can be expected when the system is put into operation. However, these results are limited to the measured operation conditions, and an important extension of the current work would involve detailed parametric studies, using a simulation approach that allows including scenarios of different bus types, propagation conditions, traffic conditions, and operation environments (e.g., dense, city, or rural areas). Using simulations would allow one to perform experiments under the controlled settings and investigate the edge cases, e.g., when one of the estimators is performing.

Another interesting aspect for future work would be to test the proposed information, fusion on different data sets, potentially extending it to work with other estimation methods, such as floor-based sensing. There could be a potential to combine data from a WLAN probe-based estimator and pressure mat estimator, since we anticipate that the error behavior is different for these sensor measurements. A combination with a video-based solution to passenger counting would likely require another information fusion approach, since the errors made by cameras for people recognition are not additive in nature.

## Figures and Tables

**Table 1:** Overview of data sets used in this work.

Data Set Tag	Day	WLAN	Light Sensor	Ground Truth
DS1	1		x	x
DS2	1		x	x
DS3	2	x		x
DS4	2	x		x
DS5	3	x	x	x
DS6	3	x	x	x

**Table 2:** Evaluation of improvement of the error between the WLAN estimator and GT after alignment of the WLAN estimator.

Data Set	Avg. MSE Org.	Avg. MSE Align.	% Improvement
DS3	150.96	99.46	34.1
DS4	34.67	23.51	32.2

## References

[1] Kte’pi B. (2014). Automated Passenger Counters. Encyclopedia of Transportation: Social Science and Policy.

[2] Kimpel T., Strathman J., Griffin D., Callas S., Gerhart R. (2003). Automatic Passenger Counter Evaluation: Implications for National Transit Database Reporting. Transp. Res. Rec. J. Transp. Res. Board.

[3] Mikkelsen L., Buchakchiev R., Madsen T., Schwefel H.P. Public transport occupancy estimation using WLAN probing. Proceedings of the 2016 8th International Workshop on Resilient Networks Design and Modeling (RNDM).

[4] Lars Mikkelsen H.P.S., Madsen T. Sensing Quality and Estimation of Public Transport Occupancy During Live Operation. Proceedings of the 2018 IEEE 17th International Symposium on Network Computing and Applications (NCA).

[5] Mikkelsen L., Schwefel H.-P., Madsen T., Burggraf A. (2022). Live Deployment Data of Bus Occupancy. https://vbn.aau.dk/da/datasets/live-deployment-data-of-bus-occupancy.

[6] Danielis P., Kouyoumdjieva S.T., Karlsson G. UrbanCount: Mobile crowd counting in urban environments. Proceedings of the 2017 8th IEEE Annual Information Technology, Electronics and Mobile Communication Conference (IEMCON).

[7] Yu H., He Z., Liu J. A vision-based method to estimate passenger flow in bus. Proceedings of the 2007 International Symposium on Intelligent Signal Processing and Communication Systems.

[8] Bernini N., Bombini L., Buzzoni M., Cerri P., Grisleri P. An embedded system for counting passengers in public transportation vehicles. Proceedings of the 2014 IEEE/ASME 10th International Conference on Mechatronic and Embedded Systems and Applications (MESA).

[9] Tu Y., Yang J. Analysis and forecast of passenger flow based on public transportation IC card and GPS data. Proceedings of the 2016 5th International Conference on Computer Science and Network Technology (ICCSNT).

[10] Zhang J., Shen D., Tu L., Zhang F., Xu C., Wang Y., Tian C., Li X., Huang B., Li Z. (2017). A Real-Time Passenger Flow Estimation and Prediction Method for Urban Bus Transit Systems. IEEE Trans. Intell. Transp. Syst..

[11] Vidyasagaran S., Devi S.R., Varma A., Rajesh A., Charan H. A low cost IoT based crowd management system for public transport. Proceedings of the 2017 International Conference on Inventive Computing and Informatics (ICICI).

[12] Pinna I., Dalla Chiara B. (2010). Automatic passenger counting and vehicle load monitoring. Ing. Ferrov..

[13] Chaudhary M., Bansal A., Bansal D., Raman B., Ramakrishnan K.K., Aggarwal N. (2016). Finding Occupancy in Buses Using Crowdsourced Data from Smartphones. Proceedings of the 17th International Conference on Distributed Computing and Networking (ICDCN ’16).

[14] Ng Y., Pei Y., Jin Y. Footfall Count Estimation Techniques Using Mobile Data. Proceedings of the 2017 18th IEEE International Conference on Mobile Data Management (MDM).

[15] Oransirikul T., Nishide R., Piumarta I., Takada H. Feasibility of Analyzing Wi-Fi Activity to Estimate Transit Passenger Population. Proceedings of the 2016 IEEE 30th International Conference on Advanced Information Networking and Applications (AINA).

[16] Pattanusorn W., Nilkhamhang I., Kittipiyakul S., Ekkachai K., Takahashi A. Passenger estimation system using Wi-Fi probe request. Proceedings of the 2016 7th International Conference of Information and Communication Technology for Embedded Systems (IC-ICTES).

[17] Bai L., Ireson N., Mazumdar S., Ciravegna F. (2017). Lessons Learned Using Wi-fi and Bluetooth As Means to Monitor Public Service Usage. Proceedings of the 2017 ACM International Joint Conference on Pervasive and Ubiquitous Computing and Proceedings of the 2017 ACM International Symposium on Wearable Computers (UbiComp ’17).

[18] Kang L., Qi B., Banerjee S. (2016). A Wireless-Based Approach for Transit Analytics. Proceedings of the 17th International Workshop on Mobile Computing Systems and Applications (HotMobile ’16).

[19] Handte M., Iqbal M.U., Wagner S., Apolinarski W., Marrón P.J., Navarro E.M.M., Martinez S., Barthelemy S.I., Fernández M.G. Crowd Density Estimation for Public Transport Vehicles. Proceedings of the EDBT/ICDT Workshops.

[20] Schauer L., Werner M., Marcus P. (2014). Estimating Crowd Densities and Pedestrian Flows Using Wi-fi and Bluetooth. Proceedings of the 11th International Conference on Mobile and Ubiquitous Systems: Computing, Networking and Services (MOBIQUITOUS ’14).

[21] Jell T., Baumgartner C., Bröring A., Mitic J., Ahram T., Taiar R., Colson S., Choplin A. (2019). BIG IoT—Interconnecting IoT Platforms from Different Domains—Final Results. Advances in Intelligent Systems and Computing, Proceedings of the International Conference on Human Interaction and Emerging Technologies (IHIET 2019), Nice, France, 22–24 August 2019.

[22] Mikkelsen L., Madsen T., Schwefel H.P. (2019). Accurate Bus Occupancy Estimation for WLAN Probing Utilizing Probabilistic Models. Int. J. Sens. Netw..

[23] Statista (2017). Number of Smartphone Users in Germany 2015 to 2022. https://www.statista.com.

[24] Statista (2017). Germany: Total Population from 2010 to 2022. https://www.statista.com.

[25] DILAX Intelcom GmbH (2018). DILAX. https://www.dilax.com/en/products/visitor-counting.

